# SELF-ACCEPTANCE BUFFERS NEGATIVE SOLITUDE-PHYSICAL ACTIVITY LINKS IN OLD AGE

**DOI:** 10.1093/geroni/igz038.3416

**Published:** 2019-11-08

**Authors:** Jody Mielcarski, Peter Graf, Maureen C Ashe, Christiane A Hoppmann

**Affiliations:** University of British Columbia, Vancouver, British Columbia, Canada

## Abstract

Loneliness is positively associated with a number of negative psychological and health outcomes. Solitude, a related yet distinct phenomenon, can have positive or negative ramifications depending on the context. As older adults spend significant time in solitude, there is particular need to investigate the effects of solitude on the health of this specific segment of the population. This study investigated everyday life associations between solitude and obstacles to physical activity as well as resources for overcoming these obstacles in order to determine whether and for whom solitude is negatively or positively associated with physical activity. Multilevel modelling was used to analyze data from 138 community-dwelling adults aged 65 years and older. Participants completed three daily questionnaires over a period of ten days concerning social context, activities and obstacles, as well as managing obstacles. Preliminary analyses using a subset of participants with complete data (N = 93) indicate that participants reported more physical activity obstacles when they were in solitude. This only applied to participants low in self-acceptance. Furthermore, self-acceptance was also positively associated with the extent to which individuals who had experienced an obstacle (N = 71) managed to overcome it. Further analyses will examine accelerometry-based movement information as well as the role of additional resources (e.g. living with others) and vulnerability factors (loneliness, anxiety).

